# Stability Analysis of a Model of Atherosclerotic Plaque Growth

**DOI:** 10.1155/2015/164035

**Published:** 2015-03-25

**Authors:** Sushruth Reddy, Padmanabhan Seshaiyer

**Affiliations:** ^1^Thomas Jefferson High School for Science and Technology, Alexandria, VA 22312, USA; ^2^Department of Mathematical Sciences, George Mason University, Fairfax, VA 22030, USA

## Abstract

Atherosclerosis, the formation of life-threatening plaques in blood vessels, is a form of cardiovascular disease. In this paper, we analyze a simplified model of plaque growth to derive physically meaningful results about the growth of plaques. In particular, the main results of this paper are two conditions, which express that the immune response increases as LDL cholesterol levels increase and that diffusion prevails over inflammation in a healthy artery.

## 1. Introduction

Atherosclerosis is a type of cardiovascular disease characterized by the accumulation of fatty deposits within the arterial intima, which can eventually expand into the lumen and obstruct blood flow. Stiff plaques are susceptible to rupture due to blood shear stress, leading to the formation of blood clots which can block the lumen entirely and lead to myocardial infarction (heart attack) [1]. According to the Center of Disease Control, cardiovascular disease is the leading cause of death in America, leading to 1 in every 4 deaths. Furthermore, 49% of all Americans possess at least one of the three major risk factors for cardiovascular disease: smoking, high low density lipoprotein (LDL) levels, and high blood pressure [2]. Thus, examining which factors in the plaque formation process are most important in causing plaque growth is of utmost importance in being able to treat patients with cardiovascular disease.

Atherosclerosis can be modeled as a multistage disease, starting with inflammation, leading to plaque growth and finally to plaque rupture and cardiac infarction [3]. Herein, we focus on inflammation and deem that plaques are stable if the inflammation cascade culminates in a return to the equilibrium state.

In this work, we study two types of lipids: high density lipoproteins (HDL) and low density lipoproteins (LDL). Both are composed of dense lipids covered by a surface protein, with molecules such as vitamin E attached for defensive purposes (3–15 for LDL molecules, 0-1 for HDL molecules) [4]. When free radicals come in contact with HDL or LDL molecules, they preferentially oxidize the vitamin E molecules attached to the protein coat. When these outer molecules have already all been oxidized, the core is oxidized. This forms oxidized LDL (ox-LDL), which acts as an irritant to the body, as well as oxidized HDL (ox-HDL), which does not seem to have any adverse effects [3]. However, HDL molecules are six times more prevalent than LDL molecules in the bloodstream and can thus act as a buffer against the buildup of ox-LDL [4].

The presence of ox-LDL causes endothelial cells to release monocyte chemoattractant protein (MPC-1), which attracts monocytes into the intima. These monocytes mature into macrophages, which phagocytize ox-LDL molecules [5]. These macrophages lose motility after having ingested the offensive molecules and turn into so-called foam cells [6]. Foam cells then cause positive feedback, releasing chemokines that lead to the recruitment of more macrophages [5]. Prompted by growth factors emitted by macrophages, smooth muscle cells from the media migrate into the intima [3].

Foam and smooth muscle cells within the intima eventually necrotize, breaking apart into detritus that coagulates into the fatty core of a plaque. This core is covered by a brittle cap of extracellular matrix (ECM) excreted by smooth muscle cells. As the plaque expands into the artery over time, blood stress on these types of plaques, called thin-capped fibroatheromas, along with the degrading effects of matrix metalloproteinases produced by macrophages, causes the plaque to rupture. The rupture of plaque releases blood clotting factors, with blood clots potentially blocking all blood flow and causing heart attack and death. Rupture, rather than complete blockage of the artery, is estimated to cause a vast majority of heart attack cases [6].

From a mathematical standpoint, much work has been done in creating realistic simulations of plaque formation via systems of partial differential equations, with novelties including different types of grids [6], inclusion of many different types of molecules [5], and the inclusion of fluid structure interaction [7].

However, comparatively little has been done in the way of mathematical stability analysis. El Khatib et al. studied a system of two partial differential equations in 2 dimensions and showed that small deviations from equilibria can lead to travelling wave solutions leading to runaway inflammation [8]. Ibragimov et al. linearized their system of differential equations and developed an ansatz based on the eigenfunctions of the von Neumann problem to find specific conditions for stability [9]. They found that the equilibrium point of the system is unstable when disease effects dominate diffusion. The primary drawback of this approach is that the ansatz relies on certain initial conditions and thus does not fully capture the dynamics of plaque growth.

Energy estimate analyses are more applicable in proving stability for large systems of differential equations, where relying on an ansatz can become unwieldy. Ibragimov et al. performed an energy stability analyses in [10, 11], respectively, and derived conditions sufficient for linearized stability. However, this approach entails the imposition of a large number of conditions, many of which can not be readily interpreted physically. The main novelty of this work is to consider a somewhat simplified differential equation system that still captures the key aspects of the plaque formation process and to derive 2 simple and sufficient criteria for linear stability.

The presentation of this work is as follows. Governing differential equations for a model of plaque growth along with the formulation of the computational domain are outlined in Section 2.  Section 3 presents a rigorous stability analysis for the linearization of the differential equations derived as well as sufficient conditions for the system equilibrium to be asymptotically stable.  Section 4 then numerically validates the criteria found in Section 3.

## 2. Mathematical Modeling

### 2.1. Domain of Interest

**Figure 1:**
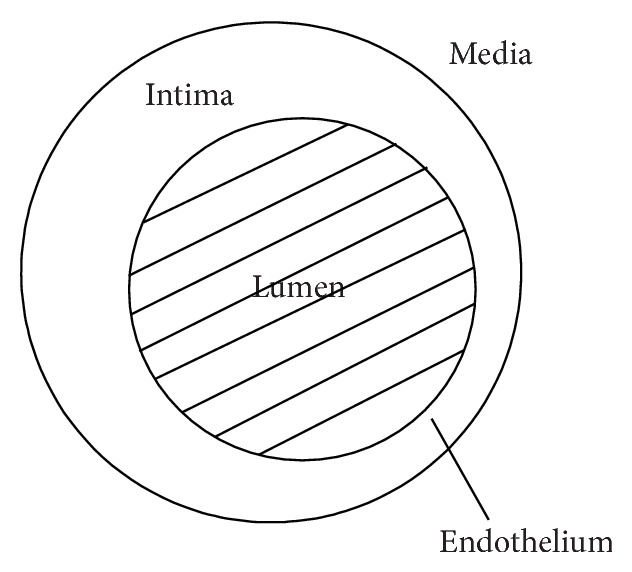
Computational domain.

Here, we study the evolution of species concentrations in the intima, as pictured in Figure 1. We assume that the intima is of fixed volume. This is a reasonable assumption as we are studying perturbations from equilibrium, more likely when total plaque growth is limited to relatively small fibrous atheromas, which do not extend extremely far into the intima. As in [9], we take the intima to be approximately shaped like a thin annulus, with some intimal thickening. We assume the outer boundary of the intima (separating it from the media) is impermeable to all chemical species.

### 2.2. Modeling Assumptions

We make a number of simplifying assumptions to obtain a differential equation system capturing the key aspects of the plaque formation process. The most important aspect is that we only consider the species LDL/ox-LDL (*L*), macrophages (*M*), and necrotic lipids (*N*). We assume that all LDL molecules entering the intima are quickly oxidized, becoming ox-LDL. We also assume that monocytes entering the intimal region quickly mature into macrophages. We assume that macrophages are attracted directly to the presence of ox-LDL molecules. We assume that the time span between the creation and eventual necrosis of foam cells is relatively short in comparison to the length of the disease progression. Finally, we assume that neutrophils and other immune cells clear lipid accumulation at a rate proportional to the concentration of lipids present.

### 2.3. Governing Differential Equations

We model plaque growth with a system of reaction-diffusion equations of the form

(1) $$\begin{array}{c} \frac{\partial f}{\partial t}+\nabla \cdot {\vec{J}}_{f}=Q_{f}. \end{array}$$

We assume that the concentration flux for species *f* is of the form ${\vec{J}}_{f}=f\nabla (\mu_{f}g)-\nabla (D_{f}f)$ (where *f* can represent LDL/ox-LDL (*L*), macrophages (*M*), and necrotic lipids (*N*)) with terms addressing the effects of Keller-Segel chemotaxis (towards species *g*) and Fickian diffusion, respectively. *D*_*f*_ is the coefficient of diffusion, and *μ*_*f*_ is the chemotactic sensitivity coefficient of *f* towards *g* [10]; both are assumed to remain constant for any fixed species across the domain due to relative intimal homogeneity. Macrophages are the only species considered that responds to chemotactic influences, so we assume that *μ*_*L*_ = *μ*_*N*_ = 0. Necrotic lipids are assumed to be immobile due to their dense structure, and so we take *D*_*N*_ = 0.

The principal contributor to the reaction terms is the reaction *L* + *M* → *N*. We make the simplifying assumption that this reaction is 1st order in each reactant (i.e., that the rate of formation of necrotic lipids ∝*LM*), with reaction rates of *k*_*L*_ and *k*_*M*_. We also assume that the rate of formation of lipids is the same as the rate of destruction of macrophages (i.e., that LDL compose an insignificant portion of the lipid mass) and that neutrophils are already dispersed throughout the intima and will destroy plaque at a rate proportional to the amount present with reaction rate *k*_*N*_.

Finally, for mathematical simplicity, we impose no-flux conditions on the boundary. As in [10], we instead add source terms to our system to represent LDL and macrophage inputs into the system. We model LDL molecule input to be constant at a rate of *L*_0_ and consider a variable influx of macrophages depending on the LDL concentration: *f*(*L*), where *f* is sigmoidal to account for saturation as the LDL concentration increases without bound.

With the above considerations, we have the following governing differential equation system:

(2) $$\begin{array}{c} \frac{\partial L}{\partial t}=D_{L}\nabla^{2}L-k_{L}LM+L_{0},\\ \frac{\partial M}{\partial t}+\nabla \cdot \left(\mu M\nabla L\right)=D_{M}\nabla^{2}M-k_{M}LM+f\left(L\right),\\ \frac{\partial N}{\partial t}=k_{M}LM-k_{N}N. \end{array}$$

## 3. Stability Analysis

In order to study the asymptotic stability of system equilibria, we linearize our system, introducing the equilibrium values of all reactants in the process, *L*_*e*_, *M*_*e*_, and *N*_*e*_. We let

(3) $$\begin{array}{c} L=L_{e}+a,\;\;M=M_{e}+b,\;\;{N=N}_{e}+c, \end{array}$$

where the equilibrium values, in terms of system parameters, are

(4) (Le,Me,Ne) =f−1kML0kL,L0kLf−1kML0/kL,L0kMkLkN,

for perturbations *a*, *b*, and *c*. We now substitute these into (2), effecting a multivariate taylor series expansion of the different terms and preserving solely linear terms (it can be seen that the constant terms cancel due to the stipulations on the values of *L*_*e*_, *M*_*e*_, and *N*_*e*_). We then obtain the system

(5) $$\begin{array}{c} \frac{\partial a}{\partial t}=D_{a}\nabla^{2}a-k_{1}a-k_{2}b, \end{array}$$

(6) $$\begin{array}{c} \frac{\partial b}{\partial t}+\chi_{a}\nabla^{2}a=D_{b}\nabla^{2}b-k_{3}a-k_{4}b, \end{array}$$

(7) $$\begin{array}{c} \frac{\partial c}{\partial t}=k_{6}a+k_{4}b-k_{5}c, \end{array}$$

where we have the parameters

(8) $$\begin{array}{c} k_{1}=k_{L}M_{e},\;\;k_{2}=k_{L}L_{e},\;\;k_{3}=k_{M}M_{e}-f^{\prime }\left(L_{e}\right),\\ k_{4}=k_{M}L_{e},\;\;k_{5}=k_{N},\;\;k_{6}=k_{M}M_{e},\\ \chi_{a}=\mu M_{e},\;\;D_{a}=D_{L},\;\;D_{b}=D_{M}. \end{array}$$

Next, we will perform a complete energy estimate analysis on our linearized system.

Our first step is to multiply the equation for all species *f* by *f* and integrate by parts over the domain using Green's identities with no-flux boundary conditions. We obtain

(9) 12∂∂t∫a2=−Da∫∇a2−k1∫a2−k2∫ab,12∂∂t∫b2=χa∫∇b·∇a−Db∫∇b2 −k3∫ab−k4∫b2,12∂∂t∫c2=k6∫ac+k4∫bc−k5∫c2.

Now we prove a lemma deriving a bound on an energy functional.

**Lemma 1 .**

Given equations (9), one has that

(10) 12∂∂t∫xa2+yb2+zc2≤k6ϵ1z+k3ϵ4y+k2ϵ5−k1x∫a2 +k4ϵ2z+k34ϵ4−k4y+k24ϵ5x∫b2 +k64ϵ1+k44ϵ2−k5z∫c2 +χaϵ3y−Dax∫∇a2 +χa4ϵ3−Dby∫∇b2.

**Proof**

In order to separate products of terms, we apply Cauchy's inequality, which states that for any *a*, *b* ∈ ℝ and *ϵ* ∈ ℝ^+^

(11) $$\begin{array}{c} \epsilon a^{2}+\frac{b^{2}}{4\epsilon }\geq ab. \end{array}$$

Thus, for the weights *ϵ*_*i*_  (*i* ∈ {1,2, 3,4, 5,6}), we have the system of inequalities:

(12) $$\begin{array}{c} \frac{1}{2}\frac{\partial }{\partial t}\int a^{2}\leq \left(k_{2}\epsilon_{5}-k_{1}\right)\int a^{2}+\left(\frac{k_{2}}{4\epsilon_{5}}\right)\int b^{2}-D_{a}\int {\left|\nabla a\right|}^{2}, \end{array}$$

(13) 12∂∂t∫b2≤(k3ϵ4)∫a2+k34ϵ4−k4∫b2 +(χaϵ3)∫∇a2+χa4ϵ3−Db∫∇b2,

(14) 12∂∂t∫c2≤(k6ϵ1)∫a2 +(k4ϵ2)∫b2+k64ϵ1+k44ϵ2−k5∫c2.

Now, we add together (12), (13), and (14), weighted by *x*, *y*, *z* ∈ ℝ^+^, respectively, to obtain the desired result.

Stability requires all perturbations to go to zero at all points in space as time increases. We first note that it is a necessary (but not sufficient) condition for lim_*t*→*∞*_∫*f* = 0 for all species *f* involved.

**Theorem 2 .**

The ordinary differential equation system associated with (5)–(7) is stable if and only if *k*_1_ *k*_4_ > *k*_2_ *k*_3_.

**Proof**

Integrating over the entire region and using the homogenous Neumann boundary conditions, we have the ordinary differential equation system

(15) $$\begin{array}{c} \frac{d}{dt}\left[\begin{array}{c} a^{r}\\ b^{r}\\ c^{r} \end{array}\right]=\left[\begin{array}{ccc} -k_{1} & -k_{2} & 0\\ -k_{3} & -k_{4} & 0\\ k_{6} & k_{4} & -k_{5} \end{array}\right]\left[\begin{array}{c} a^{r}\\ b^{r}\\ c^{r} \end{array}\right], \end{array}$$

where *f* ^*r*^ = ∫*f* for any chemical species *f*. In order for our ODE system (15) to be stable, the eigenvalues of the above matrix must all be negative [12]. The characteristic polynomial of the above matrix, *A*, is

(16) det⁡(A−λI) =−k5−λ−k1−λ−k4−λ−k2k3 =−λ+k5λ2+k1+k4λ+k1k4−k2k3.

The zeroes of this equation are *λ*_1_ = −*k*_5_, which is always negative, as well as

(17) λ2,3=−(k1+k4)±k1+k42−4(k1k4−k2k3)2=−k1+k4±k1−k42+4k2k32.

Clearly both of these zeros are real, with taking the negative sign above leading to a negative value for *λ*_2_. In order for the third zero to also be negative, we require

(18) λ3<0⟺k1+k4>k1−k42+4k2k3⟺k1k4>k2k3.

We now build up a series of lemmas in order to prove the existence of an everywhere positive energy functional that decays to zero as time increases without bound. Herein, we assume that

(19) $$\begin{array}{c} k_{1}k_{4}>k_{2}k_{3}, \end{array}$$

which follows from Theorem 2, as well as

(20) $$\begin{array}{c} \chi_{a}^{2}<\frac{4D_{b}D_{a}k_{3}}{k_{2}}, \end{array}$$

and the reasons for the sufficiency of this condition will become more apparent via the following lemmas.

**Lemma 3 .**

There exist *x*, *y*, and *z* positive such that one simultaneously satisfies

(21) $$\begin{array}{c} k_{6}\epsilon_{1}z+k_{3}\epsilon_{4}y+\left(k_{2}\epsilon_{5}-k_{1}\right)x<0,\\ k_{4}\epsilon_{2}z+\left(\frac{k_{3}}{4\epsilon_{4}}-k_{4}\right)y+\left(\frac{k_{2}}{4\epsilon_{5}}\right)x<0, \end{array}$$

where one sets *ϵ*_1_ = *k*_6_/*k*_5_, *ϵ*_2_ = *k*_4_/*k*_5_, and *ϵ*_4_ = *ϵ*_5_ = *k*_1_/2*k*_2_.

**Proof**

We may reformulate the stability condition derived in Theorem 2 as

(22) $$\begin{array}{c} \frac{2k_{1}k_{4}}{k_{2}k_{3}}>2. \end{array}$$

As all of the *k*_*i*_'s are positive, we may then write

(23) $$\begin{array}{c} 1>\frac{1}{2k_{1}k_{4}/k_{2}k_{3}-1}=\frac{2k_{3}k_{2}}{4k_{1}k_{4}-2k_{2}k_{3}}. \end{array}$$

Rearranging, we may then say that

(24) $$\begin{array}{c} \frac{k_{2}}{k_{3}}>\frac{2k_{2}^{2}}{4k_{1}k_{4}-2k_{2}k_{3}}. \end{array}$$

As we have set *ϵ*_4_ = *ϵ*_5_ = *k*_1_/2*k*_2_, this is equivalent to

(25) $$\begin{array}{c} \frac{k_{1}-k_{2}\epsilon_{5}}{k_{3}\epsilon_{4}}>\frac{k_{2}}{\epsilon_{5}\left(4k_{4}-k_{3}/\epsilon_{4}\right)}. \end{array}$$

As this inequality holds for all parameter values in our parameter space, we may choose *x* and *y* to be positive constants such that the ratio *y*/*x* lies in between these two bounds. Then,

(26) $$\begin{array}{c} \frac{k_{1}-k_{2}\epsilon_{5}}{k_{3}\epsilon_{4}}>\frac{y}{x}>\frac{k_{2}}{\epsilon_{5}\left(4k_{4}-k_{3}/\epsilon_{4}\right)}. \end{array}$$

Now, *ϵ*_5_ = *k*_1_/2*k*_2_ implies that *k*_1_ − *k*_2_ *ϵ*_5_ > 0. Similarly *ϵ*_4_ = *k*_1_/2*k*_2_ along with the stability condition derived in Theorem 2 implies that 4*k*_4_ − *k*_3_/*ϵ*_4_ > 0. This means we may multiply out terms to obtain the system of inequalities:

(27) $$\begin{array}{c} \left(k_{1}-k_{2}\epsilon_{5}\right)x>k_{3}\epsilon_{4}y,\\ \left(k_{4}-\frac{k_{3}}{4\epsilon_{4}}\right)y>\frac{k_{2}}{4\epsilon_{5}}x. \end{array}$$

Then, we have that

(28) $$\begin{array}{c} 0<\frac{\left(k_{1}-k_{2}\epsilon_{5}\right)x-k_{3}\epsilon_{4}y}{k_{6}\epsilon_{1}},\\ 0<\frac{\left(k_{4}-k_{3}/4\epsilon_{4}\right)y-\left(k_{2}/4\epsilon_{5}\right)x}{k_{4}\epsilon_{2}}. \end{array}$$

We can then choose *z* to be some positive constant less than the right hand sides of the above equation. Rearranging, we then have giving the desired result.

**Lemma 4 .**

There exists *ϵ*_3_ positive such that one can satisfy

(29) $$\begin{array}{c} \chi_{a}\epsilon_{3}y<D_{a}x, \end{array}$$

(30) $$\begin{array}{c} \frac{\chi_{a}}{4\epsilon_{3}}<D_{b}. \end{array}$$

**Proof**

From (20), we may assert the bound:

(31) $$\begin{array}{c} \frac{D_{a}k_{3}}{\chi_{a}k_{2}}>\frac{\chi_{a}}{4D_{b}}. \end{array}$$

Choosing *ϵ*_3_ such that it lies between these two bounds, we have

(32) $$\begin{array}{c} \frac{D_{a}k_{3}}{\chi_{a}k_{2}}>\epsilon_{3}>\frac{\chi_{a}}{4D_{b}}. \end{array}$$

Rearranging this inequality using the bound on *y*/*x* derived earlier gives the desired result.

The next theorem describes the conditions for asymptotic stability.

**Theorem 5 .**

The equilibrium solution (*L*_*e*_, *M*_*e*_, *N*_*e*_) is asymptotically stable given that

- *f*′(*L*_*e*_) > 0,
- *χ*_*a*_^2^ < 4*D*_*b*_ *D*_*a*_ *k*_3_/*k*_2_.

**Proof**

We define an energy functional, ℱ(*a*, *b*, *c*) = *ϕ*(*t*), equal to the left hand side of  (10). From lemmas provided earlier, we know that there exist constants *ϵ*_*i*_ and *x*, *y*, and *z* such that each of the coefficients in the definition of the functional is positive. As each of the coefficients on the right hand side is negative given the appropriate choice of constants, we may conclude for some positive *M* (the absolute value of the maximum of the coefficients of the RHS) that

(33) $$\begin{array}{c} \frac{d\phi }{dt}<-M\phi \left(t\right). \end{array}$$

This implies that the functional decays exponentially and that it is thus asymptotically stable.

The first criterion that *f*′(*L*_*e*_) > 0 is necessary for the equilibrium of the linearized system of partial differential equations to be stable. Physically, as *f* is the macrophage response to an LDL presence in the intima, this condition implies that an increased number of macrophages must permeate the intima if LDL/ox-LDL levels increase. This is characteristic of a healthy immune response, as the presence of LDL causes endothelial cells to release monocyte chemoattractant protein, attracting monocytes that later mature into macrophages [5].

The second criterion that *χ*_*a*_^2^ < 4*D*_*b*_ *D*_*a*_ *k*_3_/*k*_2_ represents loosely that the force of chemotaxis (represented by *χ*_*a*_) should be countered by diffusive forces (represented by the product *D*_*a*_ *D*_*b*_). As chemotaxis results in an aggregation of chemical species in one place, a large value of *χ*_*a*_ could result in a runaway immune cascade leading to plaque growth and eventual rupture. A strong propensity towards diffusion indicated by large values of the diffusion coefficients would temper this aggregation by opposing the concentration of species in one region.

## 4. Numerical Results

In this section, we evaluate the robustness of the stability criteria:

(34) $$\begin{array}{c} k_{1}k_{4}>k_{2}k_{3}\left(\text{equivalently}\;\;f^{\prime }\left(L_{e}\right)>0\right), \end{array}$$

(35) $$\begin{array}{c} \chi_{a}^{2}<\frac{4D_{b}D_{a}k_{3}}{k_{2}}. \end{array}$$

### 4.1. Bipolar Coordinates

Similar to [6], we implement our original differential equation system (2) in a 2-dimensional bipolar coordinate system. The conversion between bipolar and rectangular coordinates is given by

(36) $$\begin{array}{c} x=\frac{d\sinh\left(\tau \right)}{\cosh\left(\tau \right)-\cos\left(\sigma \right)},\\ y=\frac{d\sin\left(\sigma \right)}{\cosh\left(\tau \right)-\cos\left(\sigma \right)}, \end{array}$$

where curves of constant *τ* describe nonconcentric circles (with greater *τ* corresponding to smaller radius) and where the angle *σ* can vary between −*π* and *π*.

The computational domain, *τ*_*ext*_ < *τ* < *τ*_int_ and −*π* ≤ *σ* ≤ *π*, is thus an off-center annulus and so meets the requirements for our modeling assumptions to hold. We implement our equations in MATLAB using the method of lines, discretizing in the space variables *σ* and *τ* and solving a system of ordinary differential equations, with each equation corresponding to a grid point in our discretization.

Specifically, our grid points are

(37) $$\begin{array}{c} \left(\tau_{i},\sigma_{j}\right)\;\text{for}\;\;\left(i=1,2,\ldots ,m\right),\;\;\left(j=1,2,\ldots n\right),\\ \Delta \tau =\tau_{i+1}-\tau_{i}=\frac{\tau_{\mathrm{int}}-\tau_{\mathrm{ext}}}{m-1},\;\;\Delta \sigma =\sigma_{j+1}-\sigma_{j}=\frac{2\pi }{n}, \end{array}$$

where we take *σ*_*n*+*k*_ = *σ*_*k*_ for consistency.

Denoting the value of *L* at point (*τ*_*i*_, *σ*_*j*_) by *L*_(*i*,*j*)_ (and similarly for *M* and *N*), we have the system of equations:

(38) dLi,jdt=DL∇2Li,j−kLLi,jMi,j,dMi,jdt+μ∇Mi,j·∇Li,j+μMi,j∇2Li,j =DM∇2Mi,j−kMLi,jMi,j+fLi,j,dN(i,j)dt=kML(i,j)M(i,j)−kNN(i,j),

where we discretize the Laplacian operator as

(39) ∇2L(i,j)=1h(i,j)2L(i+1,j)−2L(i,j)+L(i−1,j)Δτ2vvvvvvvv+Li,j+1−2Li,j+Li,j−1Δσ2,

and similarly for *M*_(*i*,*j*)_ for (*i* = 2,3,…, *m* − 1) and (*j* = 1,2,…, *n*).

At the boundaries *τ*_int_ and *τ*_*ext*_, we employ the one-sided approximation:

(40) dL(1,j)dt=1h1,j−3F1,j+4F2,j−F3,j2Δτvvvvvvvv+G1,j+1−G1,j−12Δσ −kLL1,jM1,j,

where *F* and *G* are the *τ* and *σ* components of the flux. Due to the no-flux boundary conditions, we may assert that

(41) $$\begin{array}{c} F_{\left(1,j\right)}=F_{\left(m,j\right)}=0,\\ F_{\left(i,j\right)}=\frac{L\left(i+1,j\right)-L\left(i-1,j\right)}{2h_{\left(i,j\right)}\Delta \tau }\;\text{for}\;\;j\neq 1,m,\\ G_{\left(i,j\right)}=\frac{L\left(i,j+1\right)-L\left(i,j-1\right)}{2h_{\left(i,j\right)}\Delta \sigma }. \end{array}$$

A similar approximation scheme is used for *M* at the boundary.

### 4.2. Numerical Experiments

Setting parameter values that satisfy the second stability criterion, we first attempt to investigate the robustness of the strict first criterion. Letting

(42) $$\begin{array}{c} I\left(t\right)=\int \left(L{\left(t\right)}^{2}+M{\left(t\right)}^{2}+N{\left(t\right)}^{2}\right)dA, \end{array}$$

our previous stability results should show that *I* → *C* as *t* → *∞*, where *C* is some constant (as the perturbations from equilibrium should approach 0). Approximating this integral as a Riemann sum over grid points over time, we have numerical confirmation that this criterion holds strongly. For instance, the first two images are for *f*′(*L*_*e*_) > 0 while the latter two are for *f*′(*L*_*e*_) < 0, starting with different initial conditions.

**Figure 2:**
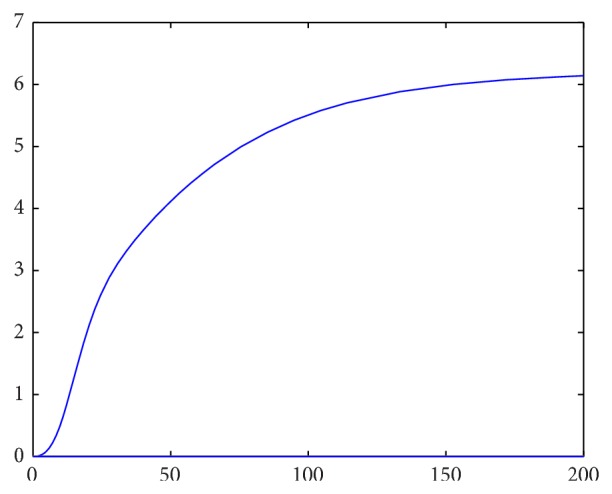
Spatial integrals of squared perturbations over time for *f*′(*L*_*e*_) > 0.

**Figure 3:**
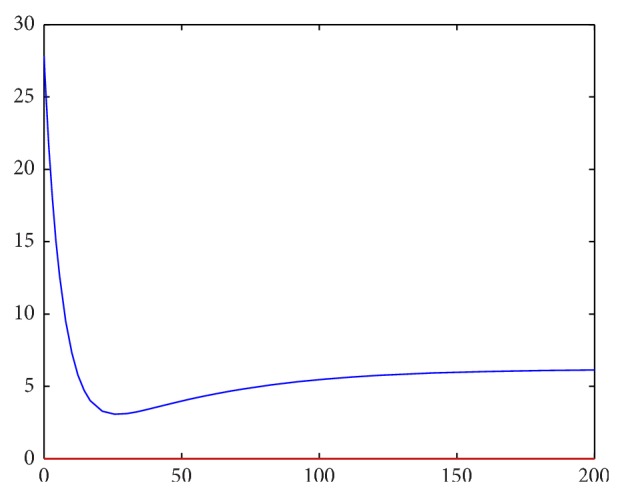
Spatial integrals of squared perturbations over time for *f*′(*L*_*e*_) > 0.

**Figure 4:**
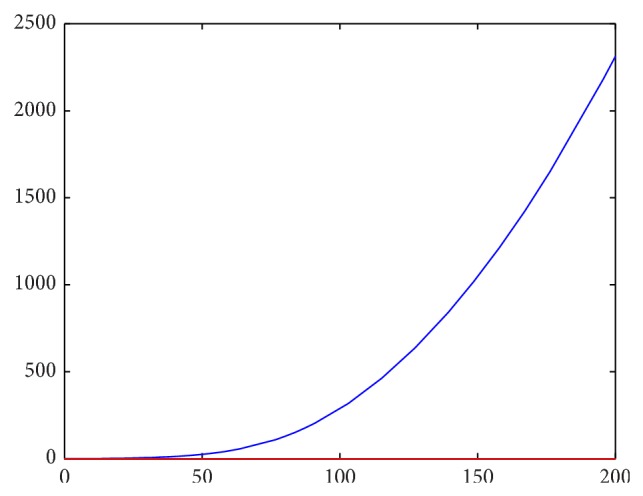
Spatial integrals of squared perturbations over time for *f*′(*L*_*e*_) < 0.

**Figure 5:**
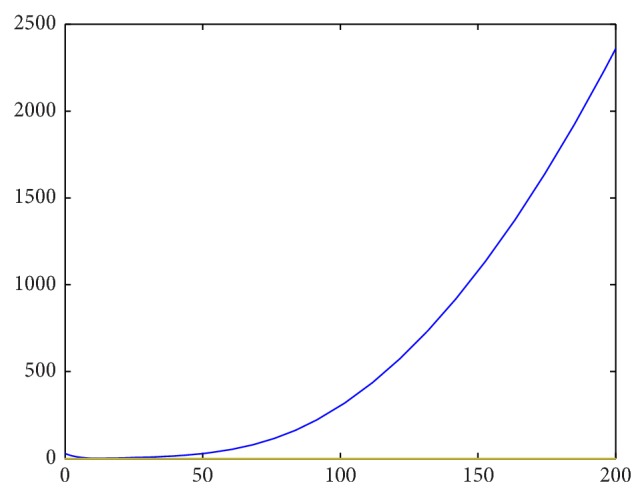
Spatial integrals of squared perturbations over time for *f*′(*L*_*e*_) < 0.

Mathematically, *f*′(*L*_*e*_) < 0 implies the existence of an eigenvalue with positive real part of the coefficient matrix *A* in the proof of Theorem 2. It is well known that this causes the space-integral of at least one of the reactants to diverge over time in the context of the linear system. *f*′(*L*_*e*_) > 0 is thus a necessary condition for stability of the linearized system and seems to also be necessary for the stability of the initial system, as illustrated by Figures 2–5.

On the other hand, setting parameter values that satisfy the first stability criterion and investigating the second stability criterion results in no such confirmation: the criterion does not seem to be necessary, only sufficient.

## 5. Conclusion

The model that we have developed encompasses the salient features of atherosclerotic plaque development, including lipid oxidation, foam cell formation, and necrosis, as well as plaque buildup. We provide sufficient conditions for a stable artery during the first inflammatory stages of atherosclerosis. Specifically, our first stability criterion reproduces the intuitive result that the influx of macrophages to phagocytize oxidized molecules should increase when the LDL concentration is increased from equilibrium in a healthy artery. The second criterion loosely says that diffusive forces must dominate inflammatory forces of chemotaxis in a healthy artery, in accordance with the results of [10]. We then numerically verify that these criteria are sufficient. A forthcoming study will include parameter estimation to increase the reliability of the model.
